# Endocrine malignancies: a still neglected issue in kidney transplantation

**DOI:** 10.3389/fmed.2025.1598168

**Published:** 2025-06-17

**Authors:** Bianca Pellegrini, Francesca Leone, Rosita Greco, Marcello Maggiolini, Michele Provenzano, Gianluigi Zaza

**Affiliations:** ^1^Department of Pharmacy, Health and Nutritional Sciences, University of Calabria, Arcavacata di Rende, Italy; ^2^Nephrology, Dialysis and Transplant Unit, “SS. Annunziata” Hospital, Cosenza, Italy

**Keywords:** endocrine malignancies, kidney transplantation, thyroid cancer, adrenal cortical carcinomas, pheochromocytomas, paragangliomas

## Abstract

Advances in kidney transplantation have made significant progress, yet challenges remain in managing both the pre- and post-transplantation phases, which have a direct impact on long-term allograft survival and comorbidities experienced by kidney transplant recipients (KTRs). Among the common immunosuppression-related complications, malignancies are a notable concern, and endocrine tumors are frequently observed. These tumors exhibit heterogeneous pathogenesis, prognosis, and treatment responses but existing literature is limited, and prevalence studies often compare KTRs to the general population. Thyroid cancers (particularly papillary thyroid cancer) have a high incidence in KTRs, whereas rare endocrine malignancies (such as neuroendocrine tumors, adrenal cortical carcinomas, pheochromocytomas, paragangliomas, and parathyroid carcinoma) are mostly reported in isolated case reports, and no clinical trials have been performed to assess the impact of different immunosuppressive treatments on their onset and development. However, current guidelines for the management of post-transplant malignancies suggest reducing or withdrawing immunosuppressive therapy whereas a switch from calcineurin inhibitors to mammalian target of rapamycin (mTOR) inhibitors is currently not recommended due to limited supporting data. Notably, the pathogenic role of transplantation and the timeline for endocrine malignancies onset in KTRs are poorly defined. To address these challenges, a multicenter and interdisciplinary approach is critical to improve our understanding of the epidemiology and pathogenesis of endocrine malignancies in KTRs. Additionally, specific guidelines for early diagnosis and treatment are necessary to ensure safe and effective management of these tumors in this vulnerable population. This mini-review aims to synthesize the available data and current insights into this important issue.

## 1 Endocrine malignancies in kidney transplant recipients: challenges and considerations

Kidney transplantation is the preferred treatment option for patients with end-stage kidney disease (ESKD), and its incidence is continually increasing (1). Based on data from 2018, the global incidence of kidney transplantation was approximately 14 per million people, and the average prevalence of ESKD requiring replacement therapy is predicted to double by 2030 (2).

Although a better overall early graft survival rate has been achieved due to significant progress in graft preservation methodologies (including machine perfusion), transplantation surgical techniques, and management/personalization of the immunosuppressive protocols, long-term allograft survival is still not optimal, and the rate of comorbidities, including tumors, remains high (3–7).

Compared with the general population, the overall risk of developing tumors is 2–4 times greater in kidney transplant recipients (KTRs), with a cumulative incidence of solid organ cancers that increases during the follow-up period, ranging from 4%−11% after 10 years (8–11) to 12%−37% 20 years after transplantation (10, 12, 13).

Multiple factors may contribute to the increased risk of tumors in this fragile patient population, including advanced recipient age, prolonged time on dialysis before transplantation (14), previous cancer, type of transplant (kidneys from deceased donors or from expanded criteria donors) (15), high reactive antibody panel (PRA) (16), acute rejection (17), and oncogenic viral infections (5, 7, 18).

Viral-associated malignancies are the most frequent type of tumor in KTRs, with a standardized incidence ratio (SIR) exceeding 11 (19). The most common types are Kaposi sarcoma (human herpesvirus 8), post-transplant lymphoproliferative disorder (PTLD; Epstein–Barr virus), hepatocellular carcinoma, and lip and anal cancers (human papillomavirus, HPV) (20).

There are also cancers that may cause ESKD and are therefore commonly seen in KTRs (e.g., myeloma and renal cell carcinoma) (21).

Additionally, non-infection-related cancers with high incidence in KTRs include non-melanoma skin cancer, non-Hodgkin lymphoma, lip and oral cavity, lung, thyroid, kidney, and prostate cancer (9, 12, 22–25).

However, the increase is not consistent across all studies or cancer sites. Some malignancies with high incidence rates in the general population, such as breast and prostate cancer, showed no increase following kidney transplantation in certain studies (7, 12, 19, 22, 26–31), while others even reported a slight decrease (9, 24, 25, 32, 33).

This discrepancy may result from variations in cancer incidence among populations, differences in data collection methods, cancer ascertainment processes, and the sex and age distribution of KTRs and the reference population (19, 34, 35).

Moreover, immunosuppression may affect the onset and development of cancer. This therapy impairs the ability of patients to control viral infections, thereby increasing the risk of infection-associated cancer. It also weakens tumor surveillance, allowing cancers to grow rapidly. However, as reported in the 2009 KDIGO guidelines, the role of these medications seems to be related to an overall inefficient immune response against tumor growth rather than the activation of specific drug-related biological/molecular mechanisms (21).

Furthermore, the degree of renal dysfunction affects cancer development in both the pre- and post-transplant periods, beginning to increase at a glomerular filtration rate of 55 ml/min and reaching a maximum three-fold increased risk with GFR ≤ 40 ml/min per 1.73 m^2^ (mainly lung and urinary) independently of other known risk factors, such as age and smoking (36).

In the presence of advanced kidney impairment, the state of chronic systemic inflammation, imbalance of oxidative stress, impairment of DNA repair, accumulation of carcinogenic compounds, excessive parathyroid hormone (PTH), and changes in intestinal microbiota contribute to tumorigenesis and are partially responsible for the increased incidence of some tumors (37).

On the other hand, we cannot rule out that the increased prevalence of some tumors (e.g., breast, prostate, lung, colorectal and thyroid cancers) in patients with ESKD could be partly due to over-diagnosis (mainly incidentally by imagine techniques) (5, 38) as well as in KTRs underlying more frequent health care access than the general population, particularly during the first year after surgery.

In fact, the American Society of Transplantation and the European Best Practice Guidelines recommend following the current cancer screening practices for common cancer types such as colorectal, breast, cervical, and prostate cancers as per the general population (39), while screening for kidney and lung cancer is not recommended (40, 41).

However, the overall incidence of endocrine and thyroid tumors in KTRs has been evaluated by several studies, which have shown a risk of up to 10 times greater than in the general population (Table 1) (7–9, 25, 27, 32, 33, 38, 42–54), but no differences when compared with ESKD patients on waiting list (23, 26, 28).

**Table 1:** Main characteristics and results of the studies included in the review.

**References**	**Years of transplantation**	**No. of KTRs**	**Region**	**Type of study**	**Median follow-up time**	**Time between transplantation and tumor diagnosis**	**Standardized incidence ratio (SIR) or incidence compared with general population**
**Endocrine tumors**							
Friman et al. (7)	1987–2016	4,514	Finland	Retrospective	9.6 years	NA	↑ (SIR 3.1)
Kasiske et al. (23)	1995–2001	35,765	USA	Retrospective	3 years	NA	↑ Relative rate vs. general population/ NSD relative risk vs. cancer in pts on waiting list
Adami et al. (28)	1970–1997	5,004	Sweden	Retrospective	NA	NA	↑ Relative risk vs. general population
Wisgerhof et al. (33)	1966–2006	1,906	Netherlands	Retrospective	9.2 years	NA	↑ (SIR 10)
**Thyroid tumors**							
Kim et al. (8)	1989–2009	2,365	Korea	Retrospective	9.8 ± 5.2 years	NA	↑ (SIR 2.5)
Villeneuve et al. (9)	1981–1998	11,155	Canada	Retrospective	Up to 19 years	NA	↑ (SIR 5)
Jeong et al. (11)	2003–2015	9,915	Korea	Retrospective	4.87 years	3.4 years	↑ (SIR 3.6)
Krynitz et al. (12)	1970–2008	7,952	Sweden	Retrospective	7.9 years	NA	↑ (SIR 4.1)
Benoni et al. (19)	1995–2011	12,984	Sweden, Norway, Denmark, and Finland	Retrospective	7 years	NA	↑ (SIR 4.24)
Lengwiler et al. (25)	2008–2014	1,557	Switzerland	Retrospective	3 years	NA	↑ (SIR 10.75)
Vajdic et al. (26)	1982–2003	10,180	Australia and New Zealand	Retrospective	8.5 years	10.7 years	↑ (SIR 6.9)
Kyllönen et al. (27)	1964–1997	2,890	Finland	Retrospective	From 0 to >10 years post–transplantation	NA	↑ (SIR 8.08)
Kyllönen et al. (28)	1970–1997	5,931	Sweden	Retrospective	6.8 years	NA	↑ (SIR 3.8)
Tessari et al. (29)	1980–2011	3,537	Italy	Retrospective	6.9 years	4.3 years	↑ (SIR 3.8)
Piselli et al. (30)	1997– 2021	11,418	Italy	Cohort study	7.1 years	NA	NSD (SIR 1.14)
Buxeda et al. (31)	1979–2014	925	Spain	Retrospective	8 years	7.4 years	↑ (SIR 2.86 in women; no change in men)
Végso et al. (32)	1973–2007	2,852	Hungary	Retrospective	94.11 months	29.9 ± 28.2 months	↑ (rate of increase) 8.95
Wisgerhof et al. (33)	1966–2006	1,906	Netherlands	Retrospective	9.2 years	NA	↑ (SIR 9.5)
Kitahara et al. (38)	1987–2012	144,276	US	Retrospective	3.9 years	NA	↑ (SIR 2.87)
Heo et al. (42)	2010–2014	1,343	South Korea	Retrospective	25.4 ± 16.7 months	27.2 months	↑ (SIR 4.58)
Hortlund et al. (43)	1977–2011	9,427 in Sweden and 4,428 in Denmark	Sweden and Denmark	Retrospective	The longest individual follow–up time was 46.8 years	NA	↑ (SIR 5.6 in Sweden population) ↑ (SIR 4.7 in Danish population)
Schrem et al. (44)	2000–2012	1,655	Germany	Retrospective	5.7 years	3.2 years	↑ (SIR 10.13)
Kim et al. (45)	2002–2017	21,191	South Korea	Retrospective	66 months	NA	↑ (SIR 3.1 in men and 2.6 in women)
Hibberd et al. (46)	1982–1997	5,970	Australia and New Zealand	Retrospective	14.2 years	NA	↑ (SIR 2.87)
Mäkitie et al. (47)	1964–1997	2,884	Finland	Retrospective	10.2 years	NA	↑ (SIR 5.8)
Hoshida et al. (48)	1970–1995	1,744	Japan	Retrospective	NA	36 months	↑ (SIR 12.43)
Birkeland et al. (49)	Up to the end of 1995	1,821	Denmark	Retrospective	7·3 years for men and 7·9 years for women	NA	↑ (SIR 10.47)
Karakose et al. (50)	1991–2020	204	Turkey	Cross–sectional	85 months	2 pts developed papillary thyroid cancer: 180 and 44 months after kidney transplantation	↑ Prevalence of thyroid nodule
Veroux et al. (51)	2000–2017	760	Italy	Retrospective	8 ± 1.2 years	5.6 years	↑ Incidence
Lee et al. (52)	1986–1999	1,739	Korea	Retrospective	137 months	NA	↑ Incidence
Karamchandani et al. (53)	2010	50,861	–	Metanalysis	8.2 years	72 months	↑ (SIR 6.9)
Pond et al. (54)	1963–2002	10,689	Australia and New Zealand	Retrospective	8.7 years for cadaveric/living unrelated donors and 7.4 years for living related donors	68 months	↑ (risk ratio 5.2)
Park et al. (55)	2007–2015	10,085	Korea	Retrospective	3.8 years	2.9 years	↑ (SIR 4.1 in men and 1.6 in women)
**Neuroendocrine tumors**							
Shah et al. (65)	1983–2020	2	UK	Observational, retrospective case series. Two kidney transplant recipients developing post–transplant cancers: a small intestine neuroendocrine neoplasm and a gastric type 3 neuroendocrine neoplasm	–	16 years for small intestine NEN and < 1 for gastric type 3 NEN	–
Karunanithi et al. (66)	2014	1	India	Case Report: kidney transplant recipient developing neuroendocrine tumor in rectum	–	8 years	–
Brady et al. (67)	2017	1 pancreas–kidney	USA	Case Report: primary small cell carcinoma of the pancreas of donor–origin	–	6 years	–
Foltys et al. (68)	2006	1	Germany	Case Report: kidney transplant recipient developing neuroendocrine carcinoma from small–cell lung carcinoma	–	1 year	–
Takeda et al. (69)	NA	2	USA	Case Report: 2 kidney transplant recipients from the same donor developing neuroendocrine carcinoma (1 in into the liver parenchyma and the other into the kidney parenchyma)	–	1 year	–
Saleeb et al. (70)	1970–2016	1,584	Canada	Retrospective	–	–	1 case of neuroendocrine tumor/carcinoid tumor arising in the appendix
**Adrenocortical carcinomas, pheochromocytomas and paragangliomas**							
Lazareth et al. (74)	2017	1	France	Case Report: paraganglioma of the bladder	–	2 years	–
Hanna-Moussa et al. (75)	2010	1	USA	Case Report: adrenal pheochromocytoma	–	–	–
Ban et al. (76)	1969–2016	3,478	Korea	Retrospective (data from 3 Korean centers): 1 case of Adrenal Cancer	–	–	–
Lo Monte et al. (77)	2009	1	Italy	Case report: oxyphil cell adrenocortical carcinoma and thyroid multifocal papillary microcarcinoma	–	7 years	–
Park et al. (78)	NA	1	Korea	Case report: adrenal cortical carcinoma and incidental renal cell carcinoma	–	15 years	–
**Parathyroid adenoma/carcinoma**							
Obregón et al. (86)	NA	1	Argentina	Case report	–	4 years	–
Kim et al. (87)	2011	1	South Korea	Case report	–	4 months	–
Evenepoel et al. (88)	1989–2004	1,743	Belgium	Retrospective: 90 patients with a functioning graft were subjected to parathyroidectomy. Histological analysis revealed 12 cases of parathyroid adenoma	62.6 months	11 months	–

SIR, standardized incidence ratio; NSD, no significant difference; NA, not available.

Although well-performed and including large patient populations, these studies have analyzed the overall prevalence of “endocrine tumors” in the absence of a clear specification of the endocrine glands involved and a clear distinction between benign tumors and histological subtypes. Therefore, this review specifically focuses on endocrine tumors in KTRs.

## 2 Thyroid malignancies

Thyroid tumors are the most extensively studied endocrine malignancies in KTRs with a high incidence (SIR >4) (Table 1) (8, 9, 11, 12, 19, 25–33, 38, 42–55). Nonetheless, a more recent Italian study, performed on a large population of ~17,000 KTRs over a 25-year period, found no difference compared to the general population (SIR 1.14) (30). The large heterogeneity in these studies is due to differences in the follow-up time, pre-transplant disease status, and post-operative immune therapy regimen.

Notably, almost all thyroid carcinomas in KTRs are differentiated microcarcinomas that are generally diagnosed incidentally, and they are almost exclusively papillary thyroid cancer (PTC) (38, 51, 53, 54, 56–58) whereas follicular carcinomas are rare (51, 57) and medullary and anaplastic carcinomas are even more rare (38).

The average time between kidney transplantation and the diagnosis of these cancers is 6 years (51, 53, 54), with a peak occurrence occurring within the first year (38, 48).

Additionally, even if there are contrasting data (38), aggressive loco-regional involvement has been reported, with the presence of lateral cervical lymph node metastases at diagnosis in almost half of the cases and variable recurrence of loco-regional disease (16.7%−75%) during a follow-up period of 94–137 months (51, 52).

Histologically solid organ transplant recipients with PTC were more likely to have multifocality and central compartment lymph node metastasis, although their tumors were smaller in size (52, 59).

It has been speculated that thyroid tumors are mainly CKD-related and dialysis-associated (with a higher incidence in KTRs and in patients with more than 5 years of dialysis vintage) (26, 38, 51).

Instead, the impact of immunosuppression on their onset and development is debated. Some authors did not find any differences in thyroid cancer incidence by immunosuppressive regimen (51, 59), although cyclosporine and azathioprine were associated with reduced thyroid cancer incidence, while tacrolimus and mycophenolate mofetil correlated with a greater risk of thyroid carcinomas, especially those with regional or distant extension (38). mTOR inhibitors (mTOR-Is) act on the primary pathogenic pathway of thyroid carcinoma (60) and could exert protective effects (61). Rapamycin-based immunosuppressive regimens in solid organ transplant recipients have been associated with a reduced incidence of thyroid cancer (38). In addition, patients with PTC undergoing this therapy showed unilateral cancer and absence of lymph node metastasis (59).

Phase 2 clinical trials have, in fact, demonstrated that oncological treatment with mTOR-Is has permitted disease stability in ~65%−76% of patients with thyroid carcinoma of different histology (62). However, no data is available on the role of mTOR-Is as immunosuppressive therapy in the incidence of thyroid malignancies in KTRs.

In a US data-linkage cohort study, the risk of thyroid cancer was increased in KTRs who underwent CD52-targeting monoclonal antibody alemtuzumab induction therapy and decreased in patients receiving muromonab-CD3 compared with those receiving no induction therapy (63). However, these results were not confirmed in the subsequent registry data analysis, which included more than 200,000 U.S. solid organ transplant recipients and 356 thyroid cancers (38).

The development of thyroid tumors is also not associated with gland functionality and the presence of anti-thyroglobulin antibodies and anti-thyroid peroxidase antibodies (50, 57) even if these studies did not differentiate benign from malignant nodules.

Further prospective studies are necessary to better define the rate of thyroid tumors in KTRs (most of them undiagnosed) in the pre- and post-transplant period, to identify the clinical, biological, and pathological fingerprints associated with their onset, and to select early diagnostic and therapeutic strategies.

In the absence of any extensive and conclusive data, the current available guidelines provide some indications for transplantation in patients on the waitlist (no waiting time for follicular/papillary tumors < 2 cm of low grade histology; at least 2 years wait for Stage 2; at least 5 years wait for Stage 3; kidney transplant contraindicated for Stage 4 or anaplastic carcinomas) (39) whereas some studies recommended specific therapeutic management of thyroid carcinomas after transplantation (64) and, as for other types of cancers, a minimization of immunosuppressive therapy. This clinical decision should take into account the risk of allograft rejection and/or severe adverse events (21). However, available case reports did not reveal significant short-time deterioration of renal function or increase of proteinuria levels after reduction of immunosuppressive therapy (50, 51). Instead, no data was provided on the long-term effect of these therapeutic changes.

Finally, in high-risk patients and those with previously recognized pre-transplant malignancy, close follow-up using specific laboratory tests and imaging technologies and monitoring of the immunosuppression therapy should be undertaken to minimize the risk of *de-novo* or recurrent cancers.

## 3 Neuroendocrine tumors

Neuroendocrine tumors (NET) are rare and most frequently occur in the intestines (65, 66), pancreas (67), lungs (68, 69), and appendix (70), accounting for 4.7% of all donor-derived cancers (71) (Table 1).

In particular, they are mostly neuroendocrine of the lung, diagnosed at a median transplant age of 10 months, with distant metastases already present at the time of diagnosis in ~73% of cases (71). This suggests the necessity to screen donors for these conditions, especially in the case of donors at high risk due to smoking, premalignant illnesses, or advanced age (72).

In a study performed on a large Canadian population of KTRs, an overall increased risk of gastrointestinal malignancies was described, with a single case of low grade neuroendocrine tumor/carcinoid tumor arising in the appendix (73).

Treatment of transmitted cancer requires graft removal and the suspension of immunosuppressive therapy, with or without chemotherapy (71).

However, some authors have suggested switching patients from calcineurin inhibitors to mTOR-Is as immunosuppression medications already employed for the treatment of well- or moderately-differentiated pancreatic NETs, G1/G2 Gastrointestinal NETs and lung NETs (65, 72, 73).

## 4 Adrenal cortical carcinomas, pheochromocytomas, and paragangliomas

Adrenal carcinomas, pheochromocytomas, and paragangliomas in KTRs are very rare disorders that have been only partially described in kidney transplantation, as in case reports, with an unclear causative role of kidney transplantation/immunosuppression (Table 1) (10, 74–78).

In an observational study performed on more than 3,700 KTRs, among the 259 cases of *de novo* malignancies, only one case of adrenocortical carcinoma was described (76).

In a case report, 79 months after the transplantation, a patient simultaneously developed papillary thyroid carcinoma and oxyphilic cell adrenal carcinoma treated with surgical eradications without adjuvant therapy (77). Another rare event is the incidental renal cell carcinoma after en-bloc resection for adrenal carcinoma (78). To minimize the risk of progression immunosuppression was minimized and mTOR-I was introduced (78).

## 5 Hyperparathyroidism and parathyroid adenoma/carcinoma

Hyperparathyroidism is a frequent complication at the time of kidney transplantation which often resolves within 1 year post-transplant. However, if persistent [particularly in KTRs with a long dialysis vintage, dysmetabolism/obesity, use of high doses of calcimimetic, high pre-transplant levels of PTH and hypercalcemia at the time of kidney transplantation (79)] it may have a significant impact on the graft function.

Patients with PTH >70 pg/ml 1 year after transplantation had a 1.37-fold higher risk of all-cause graft loss and a 1.6-fold higher risk of death-censored graft loss compared with patients without post-transplant hyperparathyroidism (80). Likewise, PTH >150 pg/ml at 3 months after kidney transplantation was an independent predictor of long-term allograft functional impairment (81).

Although the mechanisms involved in this condition are still under investigation, some studies have suggested that high levels of PTH can induce endothelial damage and cause structural vascular alterations, making those vessels less responsive to changes in blood flow and blood pressure (82, 83).

Additionally, hyperparathyroidism may induce nephrocalcinosis with consequent risk of severe impairment of graft function (84, 85).

Possible therapies for hyperthyroidism include vitamin D and vitamin D analogs, use of calcimimetics, and partial or total parathyroidectomy (39).

However, parathyroid carcinoma is rare in KTRs (86), and only a few case reports have described histological findings of parathyroid carcinoma or adenoma after parathyroidectomy for tertiary hyperparathyroidism (87, 88) (Table 1).

## 6 Conclusion

To date, a well-standardized clinical approach to endocrine tumors in KTRs remains an unmet need, and no specific guidelines have been proposed to guide the diagnosis and treatment of these conditions. In addition, the impact of immunosuppression on the onset and development of these tumors is poorly understood. Most of the studies were performed on the general population, and the small number of patients did not allow us to draw definitive conclusions.

In addition, collaborative networking (including endocrinologists, surgeons, and molecular pathologists) is needed to early identify and treat KTRs affected by these complex, often rare, cancers. Finally, larger multicenter international studies are needed to define the impact on the health system and to standardize the management of these clinical conditions in kidney transplant recipients.
